# Tick cell culture isolation and growth of *Rickettsia raoultii* from Dutch *Dermacentor reticulatus* ticks

**DOI:** 10.1016/j.ttbdis.2012.10.020

**Published:** 2012-12

**Authors:** M. Pilar Alberdi, Ard M. Nijhof, Frans Jongejan, Lesley Bell-Sakyi

**Affiliations:** a The Roslin Institute and Royal (Dick) School of Veterinary Studies, University of Edinburgh, Easter Bush, Roslin, Midlothian EH25 9RG, UK; b Utrecht Centre for Tick-borne Diseases, Faculty of Veterinary Medicine, Utrecht University, Yalelaan 1, 3584 CL Utrecht, The Netherlands; c Department of Veterinary Tropical Diseases, Faculty of Veterinary Science, University of Pretoria, Private Bag X04, Onderstepoort 0110, South Africa

**Keywords:** *Dermacentor reticulatus*, Ticks, Tick cell lines, *Rickettsia raoultii*, Endosymbionts

## Abstract

Tick cell lines play an important role in research on ticks and tick-borne pathogenic and symbiotic microorganisms. In an attempt to derive continuous *Dermacentor reticulatus* cell lines, embryo-derived primary cell cultures were set up from eggs laid by field ticks originally collected as unfed adults in The Netherlands and maintained for up to 16 months. After several months, it became evident that cells in the primary cultures were infected with a *Rickettsia*-like intracellular organism. Supernatant medium containing some *D. reticulatus* cells was inoculated into cultures of 2 *Rhipicephalus* (*Boophilus*) *microplus* cell lines, BME/CTVM2 and BME/CTVM23, where abundant growth of the bacteria occurred intracellularly on transfer to both cell lines. Bacterial growth was monitored by light (live, inverted microscope, Giemsa-stained cytocentrifuge smears) and transmission electron microscopy revealing heavy infection with typical intracytoplasmic *Rickettsia*-like bacteria, not present in uninfected cultures. DNA was extracted from bacteria-infected and uninfected control cultures, and primers specific for *Rickettsia* 16S rRNA, *ompB*, and *sca4* genes were used to generate PCR products that were subsequently sequenced. *D. reticulatus* primary cultures and both infected tick cell lines were positive for all 3 *Rickettsia* genes. Sequencing of PCR products revealed 99–100% identity with published *Rickettsia raoultii* sequences. The *R. raoultii* also grew abundantly in the *D. nitens* cell line ANE58, poorly in the *D. albipictus* cell line DALBE3, and not at all in the *D. andersoni* cell line DAE15. In conclusion, primary tick cell cultures and cell lines are useful systems for isolation and propagation of fastidious tick-borne microorganisms. In vitro isolation of *R. raoultii* from Dutch *D. reticulatus* confirms previous PCR-based detection in field ticks, and presence of the bacteria in the tick eggs used to initiate the primary cultures confirms that transovarial transmission of this *Rickettsia* occurs.

## Introduction

Tick cell lines play an increasingly important role in research on ticks and tick-borne pathogenic and symbiotic microorganisms (Bell-Sakyi et al., 2007). Primary tick tissue or organ cultures, tick cell lines, or a combination of 2 such systems have long proved to be useful for isolation of fastidious tick-borne microorganisms. For example, Řeháček and Kožuch (1964) found that primary *Hyalomma asiaticum* cell cultures were more sensitive than chick embryo cell cultures for detection of very small amounts of tick-borne encephalitis virus. Similarly, Yunker et al. (1987) used *Dermacentor variabilis* cell lines to successfully isolate spiroplasmas from field-collected *Ixodes pacificus* ticks while axenic culture in specific spiroplasma broth failed. Inoculation of infected mammalian blood cells into *I. scapularis* cell lines has resulted in isolation and continuous culture of many obligate intracellular arthropod-borne bacterial pathogens, as reviewed by Bell-Sakyi et al. (2007).

Ticks of the ixodid genus *Dermacentor* harbour several pathogenic and/or endosymbiotic *Rickettsia* species, including the human pathogens *Rickettsia rickettsii*, causative agent of Rocky Mountain spotted fever in North America, *R. slovaca* and *R. sibirica* in the Old World (Parola and Raoult, 2001), and species such as *R. montanensis* and *R. peacockii* which are not known to cause disease in vertebrates (Bell et al., 1963; Niebylski et al., 1997). Less is known about the pathogenicity of the Eurasian species *R. raoultii*, which has been found in *Dermacentor reticulatus*, *D. silvarum*, *D. nuttalli*, and *D. marginatus* ticks (Mediannikov et al., 2008), although it is considered to be an emerging pathogen and has recently been implicated as a causative agent of human tick-borne lymphadenitis (TIBOLA/DEBONEL) (Parola et al., 2009).

Currently, continuous cell lines are available from 4 New World *Dermacentor* species, some of which have been used for the isolation, propagation, and study of tick-borne *Rickettsia* spp. (Noda et al., 1997; Simser et al., 2001, 2002; Kurtti et al., 2005; Mattila et al., 2007a,b). At present, there are no cell lines available from any European or Asian *Dermacentor* species. *D. reticulatus*, also known as the European meadow tick, is found in southern, central, and, increasingly, northern Europe. Larvae and nymphs feed on rodents and adult ticks feed on dogs, cattle, sheep, and horses, and frequently attack humans (Estrada-Peña and Jongejan, 1999). *D. reticulatus* transmits canine (*Babesia canis*) and equine (*B. caballi*) babesiosis in many European countries. *D. reticulatus* ticks also transmit spotted fever group (SFG) *Rickettsia* species to humans (Stańczak, 2006) and have been shown experimentally to transmit *Anaplasma marginale* to cattle (Živković et al., 2007). In Western Siberia, *D. reticulatus* is the vector of the flavivirus causing Omsk haemorrhagic fever in humans and muskrats (Růžek et al., 2010). *D. reticulatus* ticks have been found to harbour *Borrelia burgdorferi* sensu lato, *Bartonella* spp., *Coxiella burnetii*, *Francisella tularensis*, and tick-borne encephalitis virus (Rar et al., 2005; Movila et al., 2006; Lopes de Carvalho et al., 2007; Nijhof et al., 2007a; Wójcik-Fatla et al., 2011), but their competence as a natural vector of these pathogens is unclear.

Considering the importance of *D. reticulatus* as a vector of human and livestock disease, we set up primary cultures from embryonic tissues derived from field ticks collected in The Netherlands, with a view to establishing continuous cell lines. Although unsuccessful in this aim, we were able to isolate a *Rickettsia* from the *D. reticulatus* primary cultures. Here, we describe its isolation, propagation in established tick cell lines, and partial characterisation as the SFG *Rickettsia* species *R. raoultii*, confirming previous PCR-based detection in Dutch field ticks (Nijhof et al., 2007a).

## Materials and methods

### Source of ticks and primary cell cultures

Unfed adult *D. reticulatus* ticks were collected from vegetation at ‘Dintelse Gorzen’ in Zeeland, The Netherlands. *D. reticulatus* adults were fed to repletion on a calf at Utrecht University, with approval by the Animal Experiments Committee (DEC) of the Faculty of Veterinary Medicine, Utrecht University (DEC No. 2008.II.07.068). Engorged females were sent to the University of Edinburgh where they were surface-sterilised (Bell-Sakyi, 1991) and incubated at 28 °C, 100% relative humidity, for oviposition. When the developing embryos were clearly visible in the eggs, primary cell cultures were set up from pools of 2 egg batches as described previously (Bell-Sakyi, 1991) except that flat-sided culture tubes (Nunc) were used with 2.2 ml medium per tube. Complete culture media used were either L-15, H-Lac, L-15/H-Lac, or L-15/MEM (Bell-Sakyi, 2004), and all cultures were incubated at 28 °C. The primary cultures were maintained with weekly medium changes (3/4 volume) until all the cells had died, as determined by inverted microscopic examination and/or failure to maintain medium pH which indicated cessation of metabolism.

### Tick cell lines

The *Rhipicephalus* (*Boophilus*) *microplus* embryo-derived cell lines BME/CTVM2 (Bell-Sakyi, 2004) and BME/CTVM23, a new cell line derived from a Mozambican tick strain (Nijhof et al., 2007b) by standard techniques (Bell-Sakyi, 1991), were maintained at 28 °C in L-15 (Leibovitz) medium supplemented with 10% tryptose phosphate broth (TPB), 20% foetal calf serum (FCS), 2 mM l-glutamine, 100 U/ml penicillin, and 100 μg/ml streptomycin (pen/strep). The *D. nitens* ANE58 (Kurtti et al., 1983), *D. albipictus* DALBE3 (Policastro et al., 1997), and *D. andersoni* DAE15 (Kurtti et al., 2005) embryo-derived cell lines were maintained at 32 °C in L-15B300 medium (Munderloh et al., 1999) supplemented with 10% TPB, 10% FCS, 0.1% bovine lipoprotein (MP Biomedicals), and l-glutamine and pen/strep as above. All cells used in this study were grown in 2.2 ml medium in flat-sided culture tubes, with weekly medium changes (3/4 volume).

### Light and electron microscopy

In primary cultures and cell lines, cells were resuspended by gentle pipetting and approximately 50-μl aliquots used to prepare cytocentrifuge smears. The smears were air-dried, fixed in methanol, stained with Giemsa and examined using a Zeiss Axioskop 2 Plus microscope. Photomicrographs were taken using an AxioCam MRc digital camera and software. For electron microscopy, samples of resuspended cells from *Rickettsia*-infected and uninfected control cell lines were fixed in 3% glutaraldehyde in cacodylate buffer for 2–3 h, post-fixed in 1% osmium tetroxide in cacodylate buffer, dehydrated in acetone, and embedded in Araldite resin. Sections were cut on a Reichert OMU4 ultramicrotome (Leica), stained in uranyl acetate and lead citrate, and viewed in a Phillips CM120 transmission electron microscope. Images were taken on a Gatan Orius CCD camera.

### DNA isolation

Total genomic DNA was prepared from infected and uninfected tick cells using the DNeasy Blood and Tissue Kit (Qiagen Ltd., UK) following the manufacturer's protocol. Purified DNA was eluted from the spin column with 400 μl TE buffer (2 × 200 μl) and stored at −20 °C.

### PCR amplification

A 364-bp fragment of the rickettsial 16s rRNA gene was amplified from genomic DNA using primers Rick-F1 (5′-GAA CGC TAT CGG TAT GCT TAA CAC A-3′) and Rick-R2 (5′-CAT CAC TCA CTC GGT ATT GCT GGA-3′) (Nijhof et al., 2007a). The primers 120-2788 (5′-AAA CAA TAA TCA AGG TAC TGT-3′) and 120-3599 (5′-TAC TTC CGG TTA CAG CAA AGT-3′) (Roux and Raoult, 2000) were used to amplify a 790-bp fragment of rickettsial outer membrane protein B (*ompB*). A 900-bp fragment of the cell surface antigen *sca4* gene was amplified using primers D1f (5′-ATG AGT AAA GAC GGT AAC CT-3′) and D928r (5′-AAG CTA TTG CGT CAT CTC CG-3′) (Sekeyova et al., 2001). Each 50-μl polymerase chain reaction (PCR) contained 36.85 μl molecular biology grade water (Sigma), 1 μl dNTP mix (10 mM of each dNTP), 10 μl 5× PCR buffer, 0.2 μl of each primer (100 μM), 0.4 U of Taq DNA polymerase, and 2 μl of template. Each PCR was carried out in an Applied Biosystems thermal cycler. Amplification was carried out with an initial 3-min denaturation at 95 °C followed by 40 cycles of denaturation at 95 °C for 30 s, annealing at 50 °C for 30 s, and extension at 68 °C for 1 min 30 s. The amplification was completed by holding the reaction mixture for 7 min at 68 °C to allow complete extension. The PCR products were visualised by UV illumination on a 1% agarose gel stained with ethidium bromide.

### DNA sequencing and analysis

Positive PCR products of, or close to, the expected size were purified using a QIAquick PCR purification kit (Qiagen) following the manufacturer's recommendations. DNA sequencing in the forward and reverse directions was performed by DNA Sequencing & Services, MRCPPU, College of Life Sciences, University of Dundee, Scotland, www.dnaseq.co.uk using Applied Biosystems Big-Dye Ver 3.1 chemistry on an Applied Biosystems model 3730 automated capillary DNA sequencer. Homology searches of the 16S rRNA, *ompB*, and *sca4* sequences in the NCBI database were performed using the BLAST search programme (Altschul et al., 1990). Sequences were aligned using ClustalX software (Larkin et al., 2007) for multiple sequence alignment.

### Nucleotide sequence accession number

The nucleotide sequences of the 16S rRNA, *ompB*, and *sca4* genes of the *Rickettsia* isolated from one of the *D. reticulatus* primary cultures (DRET2) have been deposited in the GenBank database and assigned accession numbers JN242190, JN242189, and JN242188, respectively.

## Results

### *D. reticulatus* primary cultures

**Fig. 1:**
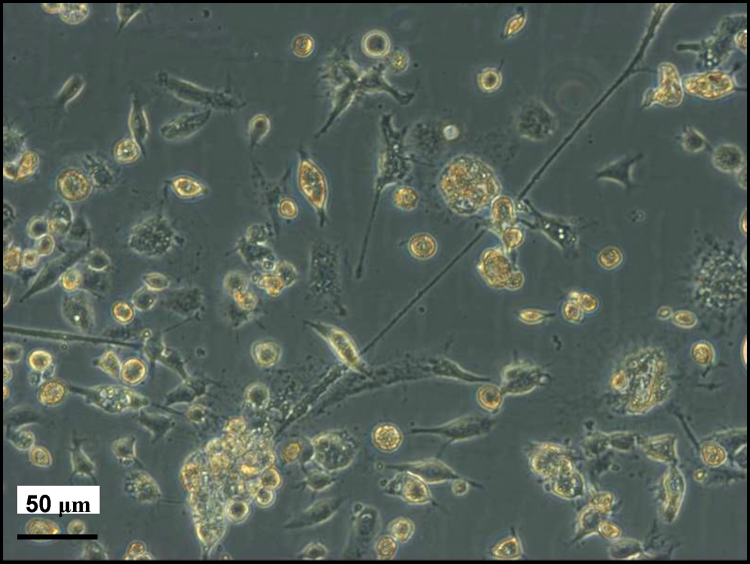
*Dermacentor reticulatus* primary embryo-derived cell culture aged 8 months. Live, phase contrast (Axio Observer inverted microscope with Axiovision software).

**Fig. 2:**
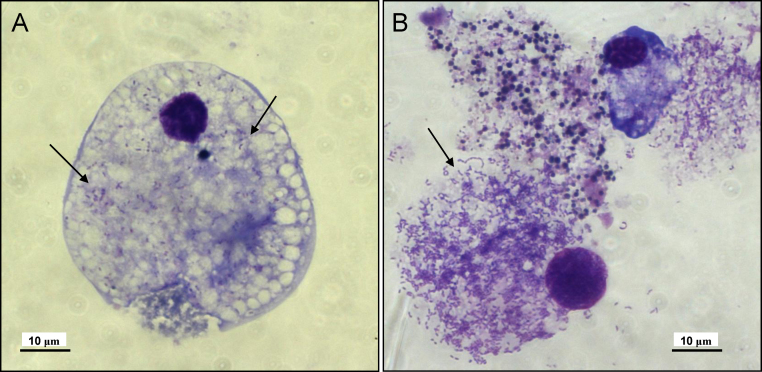
*Dermacentor reticulatus* primary cell culture infected with *Rickettsia*-like bacteria (arrows). (A) At 7 months, (B) at 12 months. Giemsa-stained cytocentrifuge smears.

Out of 15 engorged female *D. reticulatus*, only 8 laid egg batches in which some or most of the eggs were fertile. These were used in 4 pools of 2 egg batches each to set up 11 primary cell cultures (1–4 cultures per pool). Five of the primary cultures were discarded within 10 weeks due to cell death or fungal contamination. The remaining 6 cultures (Fig. 1) were maintained for up to 16 months, at which point it was evident that cell multiplication, if previously occurring, had ceased. In the 6 surviving primary cultures, cytopathic effects began to appear after 7 months in vitro; Giemsa-stained cytocentrifuge smears revealed the presence of an intracellular rod-shaped *Rickettsia*-like bacterium infecting varying proportions (<5 to >90%) of the cells in each culture (Fig. 2A). The bacteria persisted in the *D. reticulatus* primary cultures with increasing infection density, accompanied by a decrease in viable cells (Fig. 2B), until the cultures were discarded.

### Subinoculation of bacteria into tick cell lines

**Fig. 3:**
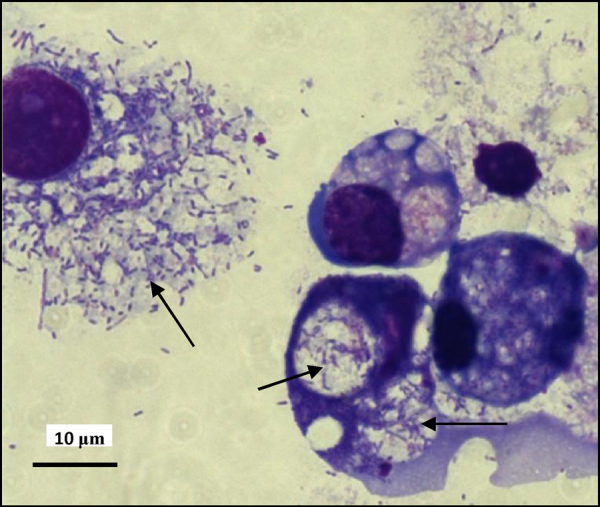
*Rhipicephalus* (*Boophilus*) *microplus* cell line BME/CTVM2 infected with *Rickettsia*-like bacteria (arrows) following subinoculation from *Dermacentor reticulatus* primary culture. Giemsa-stained cytocentrifuge smear.

At 14–16 months, 0.5 ml supernate containing a few cells from 5 of the *D. reticulatus* primary cultures was transferred onto one or more of the continuous tick cell lines BME/CTVM2 (*n* = 3) and BME/CTVM23 (*n* = 4) incubated at 28 °C, ANE58 (*n* = 3) and DAE15 (*n* = 3) incubated at 32 °C, and DALBE3 (*n* = 4) incubated at both temperatures. The bacteria grew vigorously in all BME/CTVM2 and BME/CTVM23 cultures (Fig. 3), causing host cell death within 4 weeks, and were taken through up to 4 subcultures by transferring a small amount (between 0.2 and 0.5 ml) of infected culture supernate onto fresh uninfected cells at 3–6-week intervals, with no detectable change in bacterial growth rate or infection level. The bacteria also grew vigorously in ANE58 cells, destroying the cultures within 5 weeks, but subculture was not attempted. In DALBE3 cells, a low level of bacterial infection was seen in the culture at 28 °C without any accompanying cytopathic effect, while no bacteria were detected by light microscopy at 32 °C over a 32-week observation period. No growth of the bacteria was detected in DAE15 cells over 16 weeks by light microscopy, and the cultures remained healthy.

### Ultrastructure of tick cell lines infected with *Rickettsia*-like bacteria

**Fig. 4:**
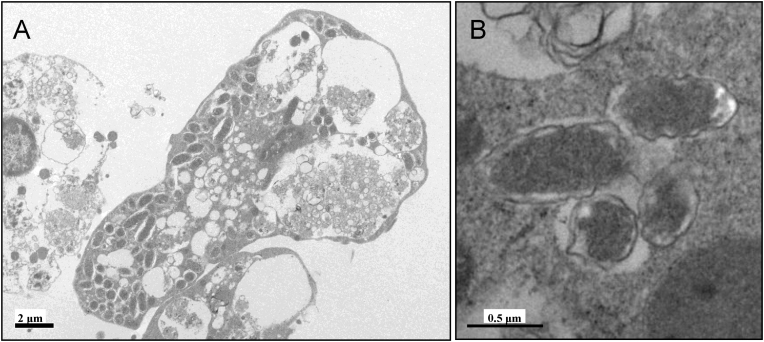
Electron micrographs of *Rickettsia*-infected *Rhipicephalus* (*Boophilus*) *microplus* cells. (A) Heavily infected BME/CTVM23 cells; (B) four *Rickettsia* in the cytoplasm of a BME/CTVM2 cell.

Samples of 2 tick cell lines, BME/CTVM2 and BME/CTVM23, both uninfected and infected with the bacteria from the *D. reticulatus* primary cultures, were examined by transmission electron microscopy. Large numbers of the bacteria were found in the cytoplasm of infected cells (Fig. 4A). The bacteria had a granular central cell body surrounded by a trilaminar cell wall typical of Gram-negative organisms. Morphologically, the intracellular bacteria resembled rickettsiae; there was a thin, electron-translucent zone outside the outer membrane of the microorganism (Fig. 4B), which is characteristic of the SFG rickettsiae (Hayes and Burgdorfer, 1982). No bacteria were seen in preparations of uninfected cell lines.

### Molecular confirmation of the identity of the intracellular bacteria as *Rickettsia raoultii*

At 14 months, DNA was extracted from samples of cell suspension from the 6 primary cultures and selected subinoculated cell lines, and PCR-screened using 3 *Rickettsia*-specific primer sets. PCR products of the expected size were amplified from all samples using primers for the 16S rRNA, *sca4*, and *ompB* genes. BLAST analysis of sequenced PCR products obtained with the *Rickettsia*-specific 16S rRNA primer set placed the bacteria in the genus *Rickettsia*. The partial *ompB* and *sca4* gene sequences were 99–100% identical to published sequences from 3 *R. raoultii* strains (Table 1). All uninfected tick cell lines were negative for *Rickettsia* with all primer sets (data not shown).

## Discussion

SFG rickettsiae are obligately intracellular, Gram-negative bacteria usually associated with ticks. Three new SFG *Rickettsia* genotypes were first identified by PCR and sequence analysis in *D. nuttalli* and *Rhipicephalus pumilio* ticks collected in Russia (Rydkina et al., 1999). These were later isolated in vitro and classified as a novel species, *R. raoultii* (Mediannikov et al., 2008). *R. raoultii* has been detected by PCR in *Dermacentor* ticks from many European countries, including The Netherlands (Nijhof et al., 2007a) and appears to be responsible for at least 8% of cases of TIBOLA/DEBONEL (Parola et al., 2009). Sequence analysis revealed that our bacterial isolate, derived from Dutch *D. reticulatus* ticks, was 99–100% identical to *R. raoultii* sequences available in the GenBank database (see Table 1).

Here, we report growth of *R. raoultii* in tick cells for the first time. Isolation from *D. reticulatus* embryos precluded the possibility that the bacteria could have been derived directly from the ticks’ previous blood meal and confirmed that transovarial transmission of *R. raoultii* occurs. We used an indirect isolation method – primary cell cultures from the vector *D. reticulatus* to amplify the bacteria, followed by subinoculation into established non-vector tick cell lines for continuous cultivation. Comparable indirect isolation methods were used to isolate *Borrelia lonestari* from *Amblyomma americanum* ticks (Varela et al., 2004), the Ap-Variant 1 strain of *Anaplasma phagocytophilum* (Massung et al., 2006), *R. monacencis* from *I. ricinus* ticks (Simser et al., 2002), and an unidentified intracellular prokaryote from *I. scapularis* ticks (Kurtti et al., 1996). In the first 2 cases, conventional isolation techniques using axenic or vertebrate cell culture systems had failed.

We found that some cell lines were more susceptible to infection with *R. raoultii* than others – in particular, lines from the 2 New World *Dermacentor* species *D. albipictus* and *D. andersoni* were poorly infected or refractory. In contrast, the *R. raoultii* was pathogenic for the 2 *Rhipicephalus* (*B.*) *microplus* lines BME/CTVM2 and BME/CTVM23, and for the *D. nitens* line ANE58. A similar pattern was seen for *R. peacockii* (Kurtti et al., 2005), which was pathogenic for the *Rhipicephalus* (*B.*) *microplus* cell line BME26, but grew poorly in DAE15 and DALBE3 cells. It is not known whether this difference in susceptibility to infection with *Rickettsia* spp. between individual cell lines is due to the species of origin or to the heterogeneous nature of uncloned, embryo-derived tick cell lines (Bell-Sakyi et al., 2007).

Previously, *R. raoultii* has been isolated and cultivated only in mammalian (L929 and Vero) cells (Mediannikov et al., 2008); the bacteria were isolated from unfed *Dermacentor* spp. ticks using the shell-vial technique (Marrero and Raoult, 1989). Our results indicate that isolation of endosymbiotic *Rickettsia* directly from ticks can also be achieved using tick cell lines. However, further experiments will be necessary to determine whether inoculation of freshly harvested embryonic tick tissues directly into a panel of potentially susceptible cell lines such as BME/CTVM2 and BME/CTVM23 will result in growth of the *Rickettsia*, or if primary cultures of the donor tick tissues are required as a preliminary amplification step.

Ticks have also been found to harbour a range of other endosymbiotic bacteria of genera including *Francisella*, *Midichloria*, *Coxiella*, *Diplorickettsia*, and *Arsenophonus* (Noda et al., 1997; Scoles, 2004; Sassera et al., 2006; Mediannikov et al., 2010, 2012). Many of these are known only through molecular analysis and have never been isolated in vitro. A similar approach to the one described in this study could be used to attempt isolation of bacteria such as the *Francisella*-like endosymbionts reported from ticks of the genera *Dermacentor*, *Amblyomma*, and *Ornithodoros* (Scoles, 2004). Moreover, there are at least 2 examples of embryo-derived tick cell lines chronically infected with rickettsiae present in the parent ticks: At passage 8 (12 months in culture), the *D. andersoni* cell line DAE100 was found to harbour *R. peacockii* (Simser et al., 2001), and *R. hoogstraali* was detected in cell lines derived from the soft tick *Carios capensis* after 6 months in vitro (Mattila et al., 2007c). Tick cell lines are usually much more permissive than mammalian cells to support *Rickettsia* replication (Simser et al., 2002; Kurtti et al., 2005). They are able to tolerate a high intracellular rickettsial burden with reduced cell lysis (Policastro et al., 1997). The availability of a system for propagation of *R. raoultii* in tick cell lines will aid in its characterisation, providing an excellent model system for study of the interaction between these bacteria and their arthropod vectors.

## Figures and Tables

**Table 1:** Level of identity of the *Rickettsia* identified in this study with sequences of *Rickettsia raoultii* strains available in the GenBank database.

	GenBank accession number/% nucleotide identity with sequence of *R. raoultii* gene		
	16S rRNA (364 bp)	*ompB* (790 bp)	*sca4* (900 bp)
*R. raoultii* strain Elanda-23/95	EU036982/100%	EU036984/100%	EU036983/99%
*R. raoultii* strain Marne (RpA4)	DQ36982/100%	DQ365797/99%	DQ365807/99%
*R. raoultii* strain Khabarovsk	DQ365810/99%	DQ365798/100%	DQ365808/99%
